# Eclectic Department

**Published:** 1854-01

**Authors:** 


Parise found in his practice the following three remedies as reliable for severe epistaxis, viz: 1. Dossils of lint, saturated with alcohol, put into the nostrils, after the patient has cleared them and cleansed them as much as possible. 2. The blowing of a powder, consisting of equal parts of alum and gum arabic, into the nostrils, and the introduction of dossils covered with the same powder. 3. The filling up of the whole cavity of the nostrils with clean cotton.—*Bulletin de Therap.*, *Tom* 13.

